# Delays in the Management of Patients with Acute Ischemic Stroke during the COVID-19 Outbreak Period: A Multicenter Study in Daegu, Korea

**DOI:** 10.1155/2021/6687765

**Published:** 2021-03-20

**Authors:** Sang-Hun Lee, You Ho Mun, Hyun Wook Ryoo, Sang-Chan Jin, Jung Ho Kim, Jae Yun Ahn, Tae Chang Jang, Sungbae Moon, Dong Eun Lee, Hyungjong Park

**Affiliations:** ^1^Department of Emergency Medicine, Dongsan Medical Center, Keimyung University, Daegu 42601, Republic of Korea; ^2^Department of Emergency Medicine, Yeungnam University College of Medicine, Daegu 42415, Republic of Korea; ^3^Department of Emergency Medicine, School of Medicine, Kyungpook National University, Daegu 41944, Republic of Korea; ^4^Department of Emergency Medicine, School of Medicine, Daegu Catholic University, Daegu 42472, Republic of Korea; ^5^Department of Emergency Medicine, Kyungpook National University Chilgok Hospital, School of Medicine, Kyungpook National University, Daegu 41404, Republic of Korea; ^6^Department of Neurology, Dongsan Medical Center, Keimyung University, Daegu 42601, Republic of Korea

## Abstract

**Background:**

Timely treatment is important for patients with acute ischemic stroke (AIS). However, the coronavirus disease 2019 (COVID-19) outbreak may have caused delays in patient management. Therefore, we analyzed the prognosis and the time spent at the prehospital and hospital stages in managing patients diagnosed with AIS during the COVID-19 outbreak.

**Methods:**

This retrospective study evaluated patients diagnosed with AIS in the emergency department (ED) at five medical centers in Daegu city between February 18 and April 17 each year from 2018 to 2020. Data on the patients' clinical features and time spent on management were collected and compared according to COVID-19 and pre-COVID-19 summaries.

**Results:**

From a total of 533 patients diagnosed with AIS, 399 patients visited the ED before COVID-19 and 134 during the COVID-19 outbreak. During the COVID-19 outbreak, compared with pre-COVID-19, AIS patients had poor National Institute of Health Stroke Scale scores at the initial hospital visit (6 vs. 4, *p*=0.013) and discharge (3 vs. 2, *p*=0.001). During the COVID-19 outbreak, the proportion of direct visits to hospitals through public emergency medical services (EMS) increased, and the onset of symptoms-to-ED door time via the public EMS was delayed (87 min vs. 68 min, *p*=0.006).

**Conclusions:**

The prognosis of AIS patients during the COVID-19 outbreak was worse than that of pre-COVID-19 patients with delays at the prehospital stage, despite the need for timely care.

## 1. Introduction

The coronavirus disease 2019 (COVID-19) caused by the novel severe acute respiratory syndrome coronavirus 2 (SARS-CoV-2) was spread worldwide in March 2020 and was declared a pandemic by the World Health Organization [1]. As of October 1, 2020, more than 30 million people have been infected with COVID-19 globally, resulting in a significant public health impact [2]. Unfortunately, the transmission route for COVID-19 is not completely understood [3]. The SARS-CoV-2 symptoms vary and are atypical, including neurological symptoms and typical fever-related respiratory symptoms [1]. Therefore, it is not easy to detect COVID-19 infection based on a patient's initial symptoms alone. Treatment of acute ischemic stroke (AIS) is extremely time-sensitive. The factors that delay treatment and reduce the critical timeframe can be related to the patient or medical system circumstances [4]. The fear of transmitting SARS-CoV-2 was very high during the COVID-19 outbreak, making patients hesitant to visit the emergency department (ED) [5]. To prevent the spread of COVID-19, many protocols are required not only in hospitals but also in the prehospital stage, causing delays in medical care [6, 7]. Thus, it is expected that the management of AIS would be affected by these changes during the COVID-19 outbreak. Some previous studies have analyzed the impact in the clinical setting; however, studies involving the prehospital stage are lacking.

**Figure 1:**
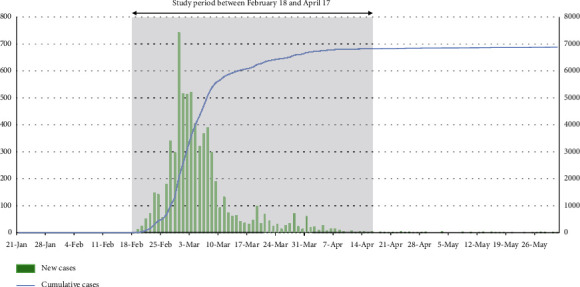
Number of COVID-19-confirmed cases in Daegu.

The first case in Daegu occurred on February 18, 2020. Since then, the number of confirmed cases has increased exponentially, and the cumulative number of confirmed cases in the first month reached 6,000. As a single city, Daegu recorded the highest rising trend and number of confirmed cases since the beginning of the pandemic at Wuhan City (Figure 1). During this period, 27 ED shutdowns occurred in six level 1 and 2 EDs in Daegu city due to quarantine failure, and each shutdown continued for approximately 16 hours. Therefore, Daegu's emergency medical system was on the verge of collapse [8]. In this study, we investigated the delay in medical care and its impact on patients with AIS in Daegu city.

## 2. Materials and Methods

### 2.1. Study Design and Search Strategy

As of December 2019, Daegu has a population of 2.47 million and is the fourth largest city in Korea. There were 16 level 3, 4 level 2, and 2 level 1 EDs, 1 fire department, and 8 fire stations, along with 48 affiliated safety centers [9]. When a rescue report is received, it is dispatched from the nearest safety center. Emergency medical personnel are transported in accordance with the first-aid instructions in the emergency room from levels 1 to 3 [10]. This retrospective study included adult patients (aged ≥18 years) diagnosed with AIS and managed in the ED from February 18, 2020, to April 17, 2020. For comparison, data were collected from patients with an ED visit diagnosed with AIS between February 18 and April 17, 2018, and 2019. The study included two of the five medical centers in Daegu city that offer essential treatment for AIS patients, including endovascular procedures. The study protocol was approved by the Institutional Review Board of the Kyungpook National University Hospital (2020-07-017) and exempted from prior consent requirements due to the retrospective nature of the study.

We screened for AIS by verifying all patients who visited the ED with an International Classification of Diseases, 10th Revision, Clinical Modification (ICD-10-CM) diagnosis of cerebrovascular disease (I60–I64). AIS was diagnosed through neurological symptoms and neuroimaging, such as computed tomography (CT) and magnetic resonance imaging, confirmed by specialists in the Department of Emergency Medicine, Neurology, Neurosurgery, and Radiology on the day of treatment [11]. In this study, patient data were collected from 2018, and patients were enrolled if they received standard care for early management within 6 hours after symptom development based on the 2015 American Heart Association/American Stroke Association guidelines [12]. The time of symptom onset was based on the first abnormal time (FAT) perceived by the patient or witness. We excluded patients if the hospital and emergency response team records did not match, refused treatment, or had already received early management treatment in other hospitals, or if the transient ischemic attack was not confirmed by objective neuroimaging. A board-certified emergency medicine doctor independently reviewed the medical records to determine the final diagnosis of AIS.

Patient data including age, sex, comorbid diseases, hospital course, mortality, and mental status scores were retrieved from the patients' electronic medical records (EMRs). The National Institute of Health Stroke Scale (NIHSS) scores were measured at the time of the visit and discharge. The type of patient transportation to the hospital and the dates associated with the development of symptoms, examinations, management, and last normal time (LNT) based on the last time the patient had no symptoms were also collected through EMRs. In the group of patients who were transported to the hospital using the public emergency medical service (EMS), data for symptom onset-to-ED door were subdivided into EMS call time, on-scene arrival time, and the first medical examination. This record was collected using an EMS runsheet.

### 2.2. Statistical Analysis

Continuous variables are reported as mean ± standard deviation or median and interquartile range. Parametric data were compared using Student's *t*-test and the Mann–Whitney *U* test for nonparametric data. Categorical variables were reported as number (%) and were compared using the *χ*2 test with Yates' correction or Fisher's exact test, as warranted. All statistical analyses were performed using IBM SPSS (version 21.0; IBM Corp., Armonk, NY, USA), with a two-sided *p* value <0.05, considered statistically significant.

## 3. Results

**Figure 2:**
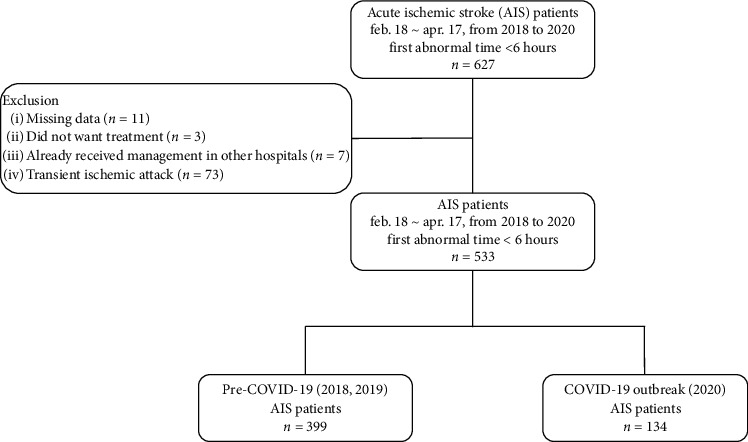
Flowchart of the study patients.

During the 2 months of 2020 selected for the study period during the COVID-19 outbreak, 134 AIS patients visited five hospitals in Daegu (DongSan Medical Center, Kyungpook National University Hospital, Yeungnam University Medical Center, Daegu Catholic University Medical Center, Daegu Fatima Hospital). During the same period in 2018 and 2019, 196 and 203 AIS patients visited the same five hospitals in Daegu (Figure 2).

For a total sample of 533 AIS patients, the mean age was 69.5 years and consisted of 326 (61.2%) men. During COVID-19, there were more direct ED visits than transfers from other hospitals (82.9% vs. 73.9%, *p*=0.022), and the patients used more public EMS during COVID than during the prepandemic period when visiting the ED (64.2% vs. 42.6%, *p* < 0.001). During in-hospital management, there was a higher rate of thrombectomy during COVID-19 and a relatively lower rate of conservative management (*p*=0.045). During the outbreak, the NIHSS scores at the ED visit and discharge were higher than in the previous years (6 vs. 4, *p*=0.013 and 3 vs. 2, respectively, *p*=0.001). During the outbreak, the mean duration of stay in the intensive care unit was longer (5.7 days vs. 4.4 days, *p*=0.047) and in-hospital mortality was higher (9.7% vs. 2.3%, *p* < 0.001) (Table 1).

The median time from symptom onset to hospital arrival was 110 min. There were no differences between symptom onset and door time, clear onset time, and time of day. In addition, the time spent on CT scans and management after arriving at the hospital was not significantly different between the two groups. During COVID-19, it took a long time from the onset of symptoms to hospital arrival for patients who moved directly to the hospital via the public EMS (87 min vs. 68 min, *p*=0.006) and no difference in timing based on other transportation methods (Table 2). The prehospital time was analyzed by segmenting it into LNT, FAT, EMS call, on-scene arrival, and door time. There was a time delay compared to pre-COVID-19 in all sections, with LNT to EMS call time (197 min vs. 70 min, *p*=0.007), FAT to EMS call (48 min vs. 31 min, *p*=0.047), response time interval (8 min vs. 7 min, *p* ≤ 0.001), and scene arrive to door time (30 min vs. 24 min, *p* ≤ 0.001) (Table 3).

## 4. Discussion

In this study, we evaluated the differences in patients diagnosed with AIS who visited the ED during the COVID-19 outbreak and before the outbreak period and identified delays in management and associated outcomes. The prognosis of AIS patients during the COVID-19 outbreak period worsened when considering neurological outcomes and mortality, but there were no significant differences observed in workups and treatments performed after arrival at the hospital. However, prehospital delays were observed in patients who were transported by public EMS during the COVID-19 outbreak.

According to a study involving two university stroke centers in Egypt, during the COVID-19 period, the patient NIHSS baseline score was higher than those pre-COVID-19 because stroke patients with minimal symptoms did not visit the hospital due to fear of coronavirus infection. The shorter transportation time and availability of caregivers to transport may be due to the curfew and lockdown, reflected in the shortened onset-to-door time [13]. However, this study lacked an accurate analysis of the time it took to transport patients. Furthermore, it had broad inclusion criteria for all stroke patients, including those with cerebral hemorrhage, and did not limit the time for onset of symptoms. To the best of our knowledge, this is the first study to analyze patients with AIS during the COVID-19 outbreak period, including prehospital factors.

The prognosis for patients during the COVID-19 outbreak period was worse than that of patients during the pre-COVID-19 period. In-hospital mortality was higher during the COVID-19 outbreak, and the neuroprognosis assessed using the NIHSS score was lower. In particular, patients with severe disease, having NIHSS scores of 21 or higher at visit, increased from 5.7% to 12.1% during the COVID-19 outbreak. In addition, patients with high NIHSS scores at discharge also increased from 6.0% to 16.7% during COVID-19. In addition, a significant difference was observed in the management of patients with AIS during COVID-19.

During the COVID-19 outbreak, the number of patients with AIS has decreased. In a previous large study, hospitalization for stroke patients was reduced by 37.9% over the last year [14]. In the present study, 134 AIS patients visited the ED during the COVID-19 outbreak, a 32.8% decrease compared to the same timeframe in 2018 and 2019. It is possible that, as risk factors for AIS decreased, the incidence decreased. For example, air pollution is a known risk factor for stroke [15]. During the COVID-19 outbreak, industrial activity was reduced, air quality was improved, and individuals were required to wear a mask when going out, reducing exposure to pollution [16]. In contrast, there may have been a reduction in the number of patients visiting the study hospitals rather than a reduction in the incidence of AIS. Since people feared being infected with COVID-19 when visiting the hospital, they hesitated to visit [14]. During COVID-19, there was a shutdown of the study hospital ED in Daegu city due to unexpected COVID-19 exposure [8]. There were restrictions on the use of hospitals by all patients, including those with AIS.

Patients with AIS who visited the ED underwent mechanical thrombectomy during the COVID-19 outbreak. Thrombectomy was performed in 13.8% of patients before the COVID-19 outbreak, but 16.4% were treated during the outbreak. Moreover, the rates of IV thrombolysis and thrombectomy together pre-COVID-19 and during COVID-19 were 8.3% to 12.7%, respectively. Although there was no significant difference in the onset-to-door time during COVID-19, more patients underwent mechanical thrombectomy. We believe this is because AIS patients with slight symptoms did not want to go to the hospital due to fear of COVID-19 infection. This study also showed that the rate of minor cases according to the NIHSS score at the time of ED visits decreased from 45.1% to 37.1%. Patients who require treatment with prominent symptoms, such as large vessel occlusion (LVO), typically visit the hospital without hesitation. As expected, a study on New York City's COVID-19 outbreak reported that many patients with LVO stroke visited the hospital [17].

The door-to-CT time, door-to-IV time, and door-to-thrombectomy time during the COVID-19 outbreak did not change from previous years. However, in the prehospital stage, differences were observed between the transfer methods, and time delays were observed. Direct hospital visits were higher during the COVID-19 outbreak, with increased public EMS utilization. AIS patients who came to the hospital using a public EMS had more time from the onset of symptoms to their arrival than during the pre-COVID-19 period. The most severe time delay was EMS call time, which may have been hesitant to use the hospital for fear of viral infection. Not only was the declaration time from the onset of symptoms delayed, but all prehospital process times were delayed, including EMS arrival at the scene and hospital arrival. Based on the recommendations of the Centers for Disease Control and Prevention's recommendation, during the COVID-19 outbreak in Daegu City, the EMS was dispatched for all calls after proper infection prevention and control practices were followed [4]. It took time to dress in protective equipment such as level D uniform, gloves, goggles, and masks, which would add to the arrival time compared to before the COVID-19 outbreak. In addition, the ED space was limited due to the temporary addition and subdivision of patients with fevers and an isolated patient zone [18]. It may have taken additional time to search for a hospital to accept patients because the EDs were running out of capacity. In addition, at all hospitals in Daegu City, a simple interview was conducted in the preliminary triage zone area to distinguish COVID-19 patients [18]. These additional processes delayed hospital examinations and treatment.

This study had several limitations. First, this was a retrospective study conducted in one city and included a relatively small number of patients. Second, the follow-up period was relatively short, and long-term results could not be determined. Third, we did not investigate the incidence of COVID-19 infection in patients with AIS or whether the virus affected stroke prognosis.

## 5. Conclusion

In conclusion, the prognosis for patients with AIS during the COVID-19 outbreak was worse than that of prepandemic patients. Despite the need for timely care, AIS patients were delayed in the prehospital stage, especially when transported through the public EMS. Appropriate measures are needed in the prehospital stage to improve the conditions for patients with AIS during the ongoing COVID-19 outbreak.

## Figures and Tables

**Table 1:** Demographic and clinical characteristics of patients diagnosed with acute ischemic stroke.

Character	Total (*n* = 533)	COVID-19 (*n* = 134)	Pre-COVID-19 (*n* = 399)	*p*
Age (yr)	69.5 ± 12.6	69.8 ± 12.1	69.4 ± 12.7	0.935
Sex, male	326 (61.2)	89 (66.4)	237 (59.4)	0.150
*Previous illness*				
Hypertension	300 (56.3)	69 (51.5)	231 (57.9)	0.197
Diabetes	146 (27.4)	41 (30.6)	105 (26.3)	0.337
Dyslipidemia	95 (17.8)	26 (19.4)	69 (17.3)	0.581
History of MI	31 (5.8)	9 (6.7)	22 (5.5)	0.607
History of CVA	111 (20.8)	33 (24.6)	78 (19.5)	0.211
Atrial fibrillation	82 (15.4)	20 (14.9)	62 (15.5)	0.865
Anticoagulation	77 (14.4)	19 (14.2)	58 (14.5)	0.919
*Visit route*				0.022
Direct	406 (76.2)	111 (82.8)	295 (73.9)	
Transfer	127 (23.8)	23 (17.2)	104 (26.1)	
*Vehicle at the time of visit*				<0.001
Public EMS	256 (48.0)	86 (64.2)	170 (42.6)	
Private EMS	101 (18.9)	20 (14.9)	81 (20.3)	
General passenger car	176 (33.0)	28 (20.9)	148 (37.1)	
*Management*				0.045
Conservative	315 (59.1)	72 (53.7)	243 (60.9)	
IV thrombolysis	91 (17.1)	23 (17.2)	68 (17)	
Thrombectomy	77 (14.4)	22 (16.4)	55 (13.8)	
IV thrombolysis and thrombectomy	50 (9.4)	17 (12.7)	33 (8.3)	
Hospital stay day	13.4 ± 15.4	15.3 ± 17.1	12.8 ± 14.8	0.249
ICU care	301 (56.5)	78 (58.2)	223 (55.9)	0.640
ICU stay day	4.7 ± 5.3	5.7 ± 5.3	4.4 ± 5.3	0.047
In-hospital death	22 (4.1)	13 (9.7)	9 (2.3)	<0.001
*Mental state*				
NIHSS at visit	*n* = 439	*n* = 124	*n* = 315	0.013
Score	4 (2–10)	6 (2–11)	4 (1–9)	
*Severity*				0.033
Minor (1–4)	188 (42.8)	46 (37.1)	142 (45.1)	
Moderate (5–15)	180 (41.0)	53 (42.7)	127 (40.3)	
Moderate to severe (16–20)	38 (8.7)	10 (8.1)	28 (8.9)	
Severe (21–42)	33 (7.5)	15 (12.1)	18 (5.7)	
NIHSS at discharge	*n* = 313	*n* = 96	*n* = 217	
Score	2 (0–5)	3 (1–9)	2 (0–5)	0.001
*Severity*				<0.001
Minor (1–4)	177 (56.5)	42 (43.8)	135 (62.2)	
Moderate (5–15)	94 (30.0)	32 (33.3)	62 (28.6)	
Moderate to severe (16–20)	13 (4.2)	6 (6.3)	7 (3.2)	
Severe (21–42)	29 (9.3)	16 (16.7)	13 (6.0)	

Data are presented as mean ± standard deviation or number (%). COVID-19, coronavirus disease 2019; MI, myocardial infarction; CVA, cerebrovascular accident; NIHSS, National Institute of Health Stroke Scale; ICU, intensive care unit; EMS, emergency medical services.

**Table 2:** The time spent on management from the symptom onset in patients with acute ischemic stroke.

Time	Total (*n* = 533)	COVID-19 (*n* = 134)	Pre-COVID-19 (*n* = 399)	*p*
Onset-to-door time	110 (61–190)	119 (71–174)	107 (58–194)	0.444
*Onset-to-door time*				0.096
<3 hours	390 (73.2)	105 (78.4)	285 (71.4)	
3–4.5 hours	89 (16.7)	20 (14.9)	69 (17.3)	
4.5–6 hours	54 (10.1)	9 (6.7)	45 (11.3)	
*Onset-to-door time on directed visit patients*				
By public EMS	*n* = 256 (48.0)	*n* = 86 (64.2)	*n* = 170 (42.6)	0.006
Onset-to-door time (min)	74 (50–127)	87 (57–143)	68 (46–114)	
By private EMS	*n* = 7 (1.3)	*n* = 1 (0.7)	*n* = 6 (1.5)	0.286
Onset-to-door time (min)	178 (126–274)	275 (275–275)	161 (114–215)	
By general passenger car	*n* = 144 (27.0)	*n* = 24 (17.9)	*n* = 120 (30.1)	0.674
Onset-to-door time (min)	135 (76–211)	134 (79–191)	135 (76–223)	
*Onset-to-door on transferred patients*				
Transfer ED visit	*n* = 127 (23.8)	*n* = 23 (17.2)	*n* = 104 (26.1)	0.376
Onset-to-door time (min)	103 (161–225)	167 (132–27)	95 (160–224)	
Door-to-CT time (min)	*n* = 505 (94.7)	*n* = 128 (95.5)	*n* = 377 (94.5)	0.903
	23 (16–32)	22 (15–35)	24 (16–32)	
Door-to-IV time (min)	*n* = 138 (25.9)	*n* = 40 (29.9)	*n* = 98 (24.6)	0.931
	47 (34–63)	47 (33–67)	47 (34–60)	
Door-to-thrombectomy time (min)	*n* = 126 (23.6)	*n* = 36 (26.9)	*n* = 87 (21.8)	0.870
	97 (69–129)	106 (66–123)	94 (69–136)	

COVID-19, coronavirus disease 2019; EMS, emergency medical services; CT, computed tomography; IV, intravenous.

**Table 3:** Prehospital transport times for public emergency medical services.

	Total	COVID-19	Pre-COVID-19	*p*
LNT-to-EMS call (min)	101 (25–362)	197 (42–544)	70 (19–330)	0.007
FAT-to-EMS call (min)	34 (10–83)	48 (14–98)	31 (9–71)	0.047
Response time interval (min)	8 (6–11)	10 (8–14)	7 (6–10)	<0.001
Scene arrival-to-door time (min)	26 (19–36)	30 (22–40)	24 (17–34)	<0.001

COVID-19, coronavirus disease 2019; LNT, last normal time; EMS, emergency medical services; FAT, first abnormal time.

## Data Availability

No data are available. The authors do not have any permission to share data on this project.
